# New Insights into the Mucus-Secreting Cells in the Proventriculus of 10 Day Old Ross 308 Broiler Chickens—A Qualitative RGB Color Study by Histochemical Assessment

**DOI:** 10.3390/life15111716

**Published:** 2025-11-06

**Authors:** Vasile Rus, Maria-Cătălina Matei-Lațiu, Adrian Florin Gal

**Affiliations:** Department of Histology, Faculty of Veterinary Medicine, University of Agricultural Sciences and Veterinary Medicine Cluj-Napoca, Calea Mănăștur No. 3-5, 400372 Cluj-Napoca, Romania; vasile.rus@usamvcluj.ro (V.R.); adrian.gal@usamvcluj.ro (A.F.G.)

**Keywords:** proventriculus, mucus secreting cells, Ross 308 broiler chickens, qualitative RGB color study by histochemical assessment

## Abstract

The proventriculus has an important adjustment function in broiler chickens, potentially impacting nutrient availability and performance. Given their contribution to protecting the gastric lining and facilitating digestion, the understanding of the mucus secretion cells within this organ is essential. Therefore, this study aims to investigate, through histochemical methods, the types of mucins secreted at the proventriculus level in 10 day old Ross 308 broiler chickens. Fragments of the proventriculus (five chickens) were histologically processed by paraffin embedding and the slides were stained using three techniques: periodic acid–Schiff (PAS) reaction, alcian blue (AB) staining pH 2.5, and combined PAS-AB staining. The color of the mucus present in the cells of the gastric mucosa was qualitatively assessed using the Photoshop Color Picker using the red, green, blue (RGB) color model and the numerical values for the RGB spectrum were analyzed from a statistical point of view. The synthesized mucus in the proventricular mucosa is predominantly PAS-positive in the superficial half of the mucosa and in the deep half, the synthesized mucins are both neutral and acidic with a predominance of acidic mucins. In the submucosa, only a few cells lining the central lumen of the glandular lobules are moderately PAS- and AB-positive.

## 1. Introduction

The poultry industry has advanced remarkably over the past 60 years. Among meat industries, poultry meat production has undoubtedly been the most successful. Production standards of broilers have continually improved over this period, with male broilers currently capable of reaching a live weight of 2.6 kg at 33–35 days of age [1]. Because of the relatively short life of a broiler chicken, most of the bird’s physiological systems, including the digestive system, are not mature even at the time of marketing. The secretion and activities of different digestive enzymes as well as nutrient transport systems vary during development after hatch. Despite the large volume of investigations conducted on aspects of early nutrition of newly hatched chicks, there remains a number of unanswered questions [2]. In some cases, the results are inconsistent, suggesting the need for further research to understand management tools to maximize resilience during the first days of life. The growth of the digestive tract occurs allometrically, with components of the digestive tract growing at different rates than the rest of the body. This growth is maximal between 4 and 8 days of age and thereafter, there is a relative decline [3].

The proventriculus, often described as the glandular stomach in birds, is situated between the esophagus and the ventriculus (muscular stomach). The proventriculus consists of four tunics: mucosa, submucosa, muscularis, and serosa [4,5]. Tunica mucosa is the innermost layer that contains mucous glands lined with simple columnar epithelial cells, containing folds that increase the surface area for secretion [4,6,7,8]. The submucosal layer encloses proventricular submucosal glands (PVSMGs) that are encapsulated by connective tissue, aiding in the secretion of digestive enzymes [9]. Tunica muscularis consists of an inner circular layer and an outer longitudinal layer of smooth muscle fibers. It aids in the mechanical mixing of food with digestive enzymes. Tunica serosa is the outermost layer, made up of loose connective tissue covered by a single layer of flattened epithelial cells [6,8].

The proventriculus plays a critical role in the avian digestive process, particularly in the initial stages of breakdown and digestion of food. By post-hatch day 1, the glands assume adult morphology and the production of neutral and acidic mucins in the stomach at embryonic day 14, reflecting the early secretory roles of glands in the stomach of broiler chickens [7]. This rapid development is crucial for early food digestion and immune defense [10].

The mucus produced in the proventriculus has several important functions. The mucous layer in the proventriculus protects the underlying epithelial cells from mechanical damage caused by food particles and digestive enzymes. It also provides lubrication, facilitating the smooth passage of food through the digestive tract [11,12]. The proventriculus secretes digestive enzymes and hydrochloric acid, which are essential for the chemical breakdown of food [9,13]. The mucus helps even in the distribution of these enzymes, ensuring efficient digestion [11,14]. Although the primary site for nutrient absorption is the small intestine, the mucus in the proventriculus aids in the initial stages of nutrient breakdown, making it easier for the nutrients to be absorbed later in the digestive process [11,12]. The mucous layer acts also as a barrier to prevent the attachment and colonization of harmful bacteria since it contains immunoglobulins such as IgA, which play a role in the immune defense by neutralizing pathogens and toxins before they can cause harm to the chicken [7,12,14,15,16].

Recent studies have revealed that mucus cells in the proventriculus vary significantly depending on the species. Regarding the histochemical profile of mucus-secreting cells, there are differences in different bird species. Thus, in pigeons, quails [6], and ducks [17], a positive reaction to Periodic acid–Schiff (PAS) staining, while in kestrels is both acidic and neutral, differs from the purely neutral secretion in domestic fowl [18]. In broiler chickens, mucus-secreting cells form tunica mucosa, secreting both neutral and acidic mucins [7], while in the excretory ducts of the glandular lobules of the submucosa, the cells are PAS-positive, suggesting that they secrete neutral mucins [19].

The anterior digestive tract, including the proventriculus, has an important adaptative function in broiler chickens when stimulated by intermittent feeding or structural components, potentially impacting nutrient availability and performance [20]. An understanding of the mucus-secreting cells within this organ is essential, given their contribution in protecting the gastric lining mucosa and in facilitating digestion. Recent studies have revealed that mucus cells in the proventriculus vary significantly in number and function, especially in young broilers. This period is particularly crucial for digestive health, as broilers undergo rapid growth and physiological changes that can impact nutrient absorption and overall performance. Investigating the histochemical properties of these mucus-secreting cells sheds light on their roles in gastrointestinal health, potentially influencing the development of dietary strategies aimed at enhancing growth and welfare in poultry. Such findings are underscored by the emerging knowledge about the effects of different feed additives on digestive health [21]. Therefore, a detailed histochemical assessment of the mucus-secreting cells is essential for enhancing poultry health and growth performance. The advantage of histochemical staining, compared to immunohistochemically ones, is its relatively low cost and the large amount of information that can be obtained with its use [22]. The available literature demonstrates that the relative growth rate of digestive organs in broilers is allometrically maximized within the first week of life and declines thereafter to eventually approach that of the gain in body weight. These findings are consistent with the demand on supply organs imposed by the maximal relative body growth being achieved from 5 to 10 days of age. At 10 days of age, the broiler chicken’s digestive tract is in a period of rapid development and maturation, moving from an immature system to one that can efficiently digest and absorb nutrients. Around days 9 and 10, the digestive organs, including the proventriculus, gizzard, small intestine, and liver, experience their highest rate of growth. The chick’s ability to efficiently digest proteins and amino acids significantly improves around the 10-day mark, emphasizing the need for highly digestible protein in starter diets [3,23]. The world-wide demand for animal feed is expected to increase to 1500 Mton in 2050. Feed formulation and production technology is gaining importance due to a number of future global opportunities, challenges, and threats. These challenges are leading to demands for innovation in a number of areas related to animal nutrition including feed technology [24].

The analysis of histochemical profiles of the proventriculus in farm chicken allows producers to make informed, data-driven improvements to their feed formulation, along with appropriate timing of feed additives and animal welfare practices. Accordingly, the study aims to investigate, through histochemical methods, the types of mucins secreted at the proventriculus level in 10 day old Ross 308 broiler chickens and perform the qualitative characterization of the color in the RGB spectrum of the mucins present in the secretory cells of the mucosa.

## 2. Materials and Methods

In this study, 5 clinically healthy Ross 308 broiler chickens (10-day-old) were euthanized and one fragment/animal from the central area of the proventriculus was harvested. The chicken raising system is on the ground, on permanent bedding, with an automatic feeding and watering line. Ventilation, temperature, humidity, and lighting are automatic. The light/dark ratio is 10 min/hour. The harvested samples were fixed with 10% buffered formalin for 5 days. At the end of the fixation period, the fragments were dehydrated with ethylic alcohol, clarified with n-butanol, and embedded in paraffin. Subsequently, 4 µm thick seriated sections were made using a Leica rotary microtome (RM 2125, by Leica Biosystems Nussloch GmbH, Nußloch, Germany) and mounted on histological slides.

To identify the types of mucins secreted in the proventriculus, 3 histochemical techniques were used: periodic acid–Schiff (PAS) reaction, alcian blue (AB) staining pH-2.5, and combined PAS-AB staining [25]. To ensure consistent cytoplasmic staining across the three techniques, and to prevent the nuclear dye from interfering with the cytoplasmic color, hematoxylin (the nuclear stain used for the PAS and PAS-AB techniques) was also used for the AB pH 2.5 staining instead of Nuclear Fast Red. The differences in background staining of the cytoplasm may negatively influence the RGB color spectrum of mucus present in the cytoplasm of cells between the three histochemical techniques used. PAS reaction was conducted to highlight the neutral mucins while AB pH-2.5 stain was used to identify the acidic mucins. The combined PAS-AB was used to show the ratio between the amounts of two types of mucins secreted by each cell. To accurately determine the ratio of acidic and neutral mucins in each examined cell, serial sections of 3 consecutive slides (4 µm thick tissue sections) were performed and stained using the 3 techniques. The histological slides were examined (by 3 examiners) under an optical microscope (Olympus BX41, Tokyo, Japan) and microphotographs were acquired (Olympus E330 camera, Tokyo, Japan). Image acquisition was performed using Olympus cellSens Entry 3.1 software, and “One Touch White Balance” along with automatic exposure was used. The light intensity under the microscope was level 4.

The color of the mucus present in the cells of the gastric mucosa was qualitatively assessed using the Photoshop Color Picker (Adobe Photoshop CS2 V9.0) using the red, green, blue (RGB) color model. The RGB analysis is a very confident way to analyze colors, as proven by other reports [26]. Briefly, since human perception of color is not linear, some studies use weighted RGB values to better align the data with human visual interpretation [26]. In Adobe Photoshop, none of the image-processing macros was used. Thus, for each staining technique (PAS, AB pH-2.5, PAS-AB), the color of the mucus in 100 cells of the gastric glands (i.e., 50 cells from the apical half and 50 cells from the basal half) was determined and the numerical values in the RGB spectrum were collected. From the acquired microphotographs, the cells assessed were the ones intercepted sagittally by the section (including the sagittal zone of the nucleus). Concerning the selection area of the stained mucus, the central zone of the mucous droplet situated in the apical pole of the cell was selected.

The numerical values for the RGB spectrum were assessed from a statistical point of view, including descriptive statistics, normality tests, and multiple comparison tests for each staining, comparing the two segments analyzed. Accordingly, the normally distributed data, according to the Kolmogorov–Smirnov test (values obtained only for PAS staining), were compared with ordinary one-way ANOVA and Tukey’s multiple comparisons test, with the significance threshold set at *p* < 0.05. For the other two stainings (AB pH-2.5 and PAS-AB), the comparisons in between the apical and basal halves were performed with Kruskal–Wallis test, followed by Dunn’s multiple comparisons test, with the same significance threshold (*p* < 0.05). All the statistical analyses were performed using GraphPad Prism 8.0.1. The confidence intervals (CIs) of the mean were calculated with a confidence level of 95%. All the statistical analyses were realized in order to provide a clear image about the mucus distribution (apical vs. basal half) at the proventriculus level, using different stainings that target different types of mucins.

The study was approved by the Bioethics Committee of the University of Agricultural Sciences and Veterinary Medicine Cluj-Napoca, no. 453 from 29 May 2024.

## 3. Results

The proventriculus in 10-day-old Ross 308 broiler chickens presents the specific structure of birds, with the four tunics present: mucosa, submucosa, muscularis externa, and serosa. In the structure of the mucosa, the surface epithelium as well as the glandular epithelium is made of simple columnar epithelium. The columnar cells have oval nuclei located in the basal half of the cell. It is found that the height of the cells is not identical. The cells on the surface and those in the apical third of the glands are approximately 2 times higher than those from the bottom of the glands. In the submucosa, the epithelium lining the central lumen of the lobules is also simple columnar epithelium and the cells have oval nuclei located approximately centrally.

**Figure 1:**
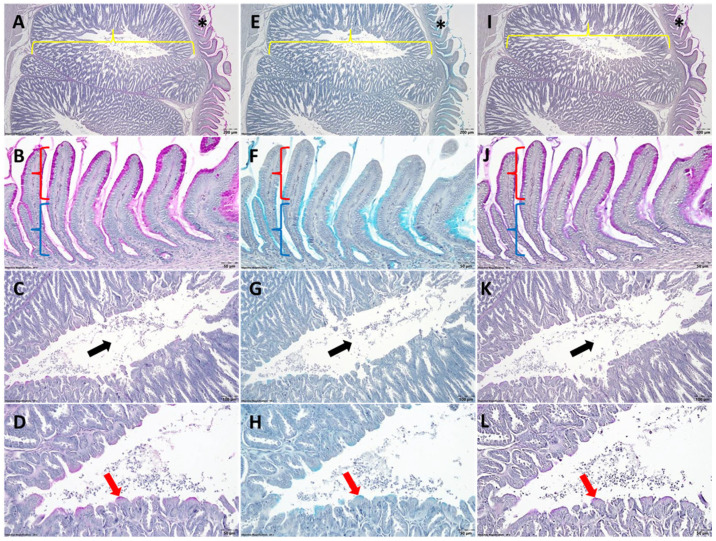
Histochemical features regarding the mucin type distribution in the proventriculus of 10 day old Ross 308 broiler chickens; (**A**,**E**,**I**) general features of the tunica mucosa and tunica submucosa; (**B**,**F**,**J**) details of the glands belonging to the tunica mucosa; (**C**,**G**,**K**) general aspects of the glandular lobules from submucosa; (**D**,**H**,**L**) details from the epithelium lining the central lumen of the glandular lobules; (**A**–**D**) PAS; (**E**–**H**) AB pH-2.5; (**I**–**L**) PAS-AB; asterisk—tunica mucosa; yellow brace—tunica submucosa; red brace—apical half of the glands of the tunica mucosa; blue brace—basal half of the glands from the tunica mucosa; black arrow—central lumen of the glandular lobules from submucosa; red arrow—the epithelium lining the central lumen of the glandular lobules.

In the PAS method, the cells in the mucosa (Figure 1A,B) displayed a positive reaction, but with a range of differences in terms of the amount of mucus stored in the cells, as well as minor variations in the intensity of the reaction. Accordingly, from the surface of the mucosa towards the bottom of the glands, both the amount of intracellular mucus and the intensity of the PAS reaction diminish.

In the submucosa, the only PAS-positive cells are those lining the central lumen of the lobules (Figure 1C). The amount of PAS-positive mucus present in the cells is very small, and most cells are PAS-negative (Figure 1D).

Concerning the AB pH-2.5 staining of the proventricular mucosa (Figure 1E), the presence of AB-positive mucous type can be observed. Most of the cells in the surface epithelium do not contain AB-positive mucus. As for the glandular epithelium, in the majority of its structure, the cells located in the middle and deep thirds contain AB-positive mucus. In a few glands, the cells in the apical half do not contain AB-positive mucus. Interestingly, the amount and the intensity of AB-positive mucus present in the cells of the glands increase gradually towards the bottom of the glands (Figure 1F).

In the submucosa, small amounts of AB-positive mucus can be observed only in the cells lining the central lumen of the lobules (Figure 1G). Most of these cells do not contain acidic mucus, whereas the AB-positive cells contain small amounts of mucus in the apical pole (Figure 1H).

Concerning the combined PAS-AB staining (Figure 1I–L), it can be observed that both the mucus-secreting cells in the mucosa and those lining the central lumen of the proventricular lobules in the submucosa secrete both PAS-positive and AB-positive mucus types, in different proportions, depending on the topographical location. Thus, the intensity of the reaction in the mucus-secreting cells located mainly in the apical half of the mucosal glands is similar to the one detected in the PAS reaction (Figure 1J).

The values recorded by the RGB color spectrum were statistically processed, and Table 1 presents the minimum value (Min), maximum value (Max), mean value (M), standard deviation (SD), standard error of the mean (SEM), and coefficient of variation (CV%), together with the lower (L 95% CI) and upper (U 95% CI) 95% CI mean.

**Figure 2:**
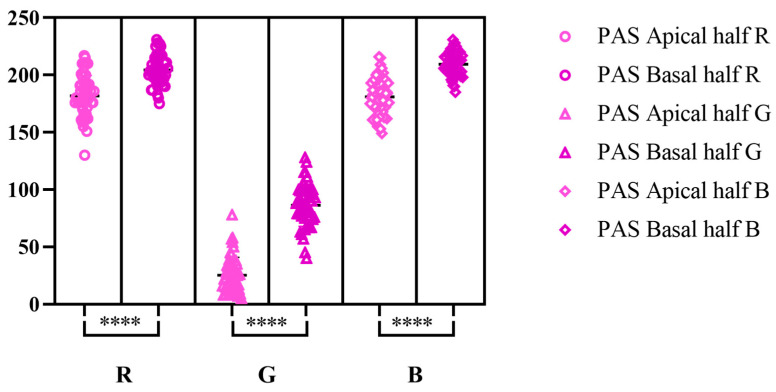
Tukey’s multiple comparison test for RGB values on PAS reaction (significance threshold set at *p* < 0.05; ****—highly significant differences); effect size (apical half R; G; B; basal half R; G; B): 181.7 (95% CI [176.7, 186.8]); 25.22 (95% CI [20.76, 29.68]); 181 (95% CI [176.5, 185.6]); 204.6 (95% CI [200.8, 208.3]); 86.44 (95% CI [81.21, 91.67]); 209.4 (95% CI [206.5, 212.3]).

**Figure 3:**
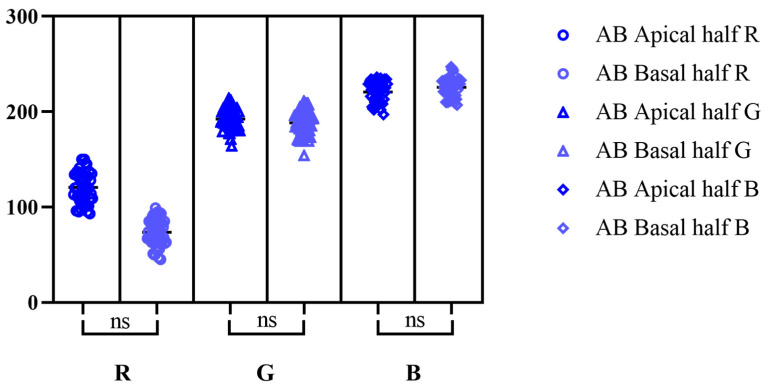
Dunn’s multiple comparison test for RGB values on AB pH-2.5 stain (significance threshold set at *p* < 0.05; ns—not significant differences); effect size (apical half R; G; B; basal half R; G; B): 120.7 (95% CI [116.0, 125.4]); 192.3 (95% CI [189.2, 195.4]); 220.5 (95% CI [176.5, 185.6]); 73.5 (95% CI [217.5, 223.5]); 188.4 (95% CI [184.8, 192.0]); 225.5 (95% CI [222.7, 228.4]).

**Figure 4:**
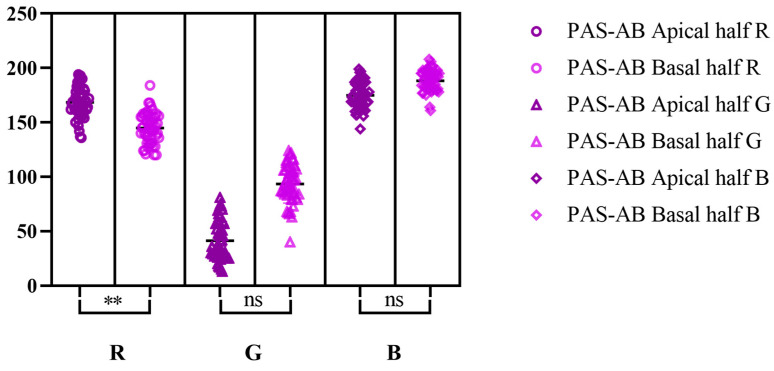
Dunn’s multiple comparison test for RGB values on combined PAS-AB stain (significance threshold set at *p* < 0.05; **—significant differences; ns—not significant differences); effect size (apical half R; G; B; basal half R; G; B): 168.5 (95% CI [164.5, 172.5]); 41.32 (95% CI [36.26, 46.38]); 175 (95% CI [171.5, 178.5]); 145 (95% CI [141.0, 149.0]); 93.54 (95% CI [88.56, 98.52]); 188.3 (95% CI [185.5, 191.1]).

According to Tukey’s multiple comparisons tests, RGB values for PAS reaction displayed significant differences in between the two halves analyzed (Figure 2). Contrary, for AB pH-2.5 staining, Dunn’s multiple comparison test showed no significant differences in between the apical half and basal one (Figure 3), while for the combined staining, the only significant difference was registered for R color from RGB spectrum (Figure 4).

The mucus synthesized by the cells in the mucosa, in the two areas compared, was qualitatively investigated using the RGB spectrum by determining the average color, the darkest color, and the lightest color across the three staining techniques used (Table 2).

## 4. Discussion

When comparing the average color of the mucus synthesized by the cells in the apical half on the PAS reaction with that from the PAS-AB staining, small differences in color shade are found, which indicates that the cells in the apical half of the glands secrete mainly neutral mucins. In contrast, the cells in the basal half of the glands secrete both types of mucins, but the amount of acidic mucins is slightly higher. Similar statistical tests to individual R, G, and B channels were made by other authors [27], which demonstrates that a single color of the RGB spectrum is sufficient to prove a major change in the structure. Accordingly, in the study performed by Kai et al. (1999) [27], it was noticed that the R and B values of eosinophilic cells were confirmed statistically by a number of values between normal, borderline atypical, and malignant squamous cells. G and B values of light green-philic cells demonstrated a statistical difference between normal, borderline atypical, and malignant squamous cells. No significant differences were found in RGB values between normal, borderline atypical, and malignant squamous orangeophilic cells [27]. As a comparison, the statistical differences between individual R, G, and B channels noticed in our study could be useful as well to prove the existence of a range of changes in the structure of the proventriculus.

Udoumoh et al. (2020) [7] state that at embryonic days 14 and 19 and post-hatch days 1, 3, and 21, the lining epithelium of the proventriculus of broiler chickens demonstrated the presence of neutral mucins, while the lining of epithelium of the proventriculus at post-hatch days 3 and 14 showed a moderate presence of acidic mucins and at embryonic day 19 and post-hatch days 1 and 3, the simple tubular glands of the lamina propria mucosae showed a moderate presence of neutral mucins [7].

In the glandular stomach of Hoopoe, the cells in the apical half of the glands in the proventriculus mucosa are predominantly AB-positive and those in the deep half are predominantly PAS-positive; in the Kingfisher, the cells in the mucosal glands synthesize both types of neutral and acidic mucins, but the amount of neutral mucin is greater in the apical and basal halves [28].

In moorhen, the cells from the apical half of the glands secrete both types of mucins, with PAS-AB staining the mucus in magenta, while in the basal half, the cells predominantly synthesize acidic mucins [29]. The aspects we observed in broiler chickens in the basal half of the glands in the proventriculus mucosa are comparable to those reported by Taher et al. (2020) [29]. However, large differences are found in the amount of mucus secreted by the epithelium lining the central lumen of the glandular lobules in the submucosa. Thus, in the study conducted by Taher et al. (2020) [29], large amounts of both neutral and acidic mucins present in the cytoplasm of the cells in the epithelium lining the glandular lobules is very small and no mucins were observed in most of the cells.

The presence of both neutral and acidic mucin types in the cells of the proventriculus mucosa was also reported in sparrow hawk, crow, and sparrow [30], poonchi bird [31], and *Coturnix coturnix* [32].

In the proventriculus of the common moorhen, the upper half of the tunica mucosa presents an AB reaction and the basal half has a PAS-positive reaction [33].

The mucus synthesized in the proventriculus has various functions, such as protecting and lubricating [11,12], distributing the enzyme synthesized [7,14], acting as a barrier against pathogens by preventing the attachment and colonization of harmful bacteria [11,16,34], and also containing immunoglobulins [15].

The design limits of the study are the low number of animals included in the report, along with the assessment of the mucous type in different ages. Furthermore, another limitation of the study could be the qualitative assessment of mucins by histochemical stains that are less efficient compared to immunohistochemistry (IHC). Still, since mucin structure and expression in chickens can differ from those of mammals, there is a limited utility of IHC in birds, with inconsistent results, a fact that proves the utility of histochemistry in non-mammalian species [35]. Another limitation of the study is that the obtained results are for 10-day-old Ross 308 broiler chickens, and our findings cannot be extrapolated to other broiler or non-broiler chicken lines.

## 5. Conclusions

The three histochemical stains suggested a slight difference between the different proventricular regions assessed. Accordingly, the synthesized mucus in the proventricular mucosa is predominantly PAS-positive in the superficial half of the mucosa, whereas in the deep half, the synthesized mucins are both neutral and acidic, with a predominance of acidic mucins. On the other hand, in the submucosa, only a few cells lining the central lumen of the glandular lobules are moderately PAS- and AB-positive. Finally, the evaluation of histochemical profiles in different segments of the digestive tract of farm broiler chickens—a species with an exceptional metabolic profile suggested by their high feed efficiency and growth rates—provides producers with the necessary data to optimize feed composition, regulate the administration timing of feed additives, and refine animal welfare protocols.

## Figures and Tables

**Table 1:** Descriptive statistics for RGB values in the two assessed regions of the proventriculus, stained with PAS, AB pH-2.5, and PAS-AB.

	PAS						AB pH-2.5						PAS-AB					
	Apical Half			Basal Half			Apical Half			Basal Half			Apical Half			Basal Half		
	R	G	B	R	G	B	R	G	B	R	G	B	R	G	B	R	G	B
**Min**	130	5	149	175	40	185	93	164	197	45	154	207	136	13	144	120	40	161
**Max**	217	78	216	231	128	231	150	214	236	99	211	247	194	81	199	184	124	208
**M**	181.7	25.22	181	204.6	86.44	209.4	120.7	192.3	220.5	73.5	188.4	225.5	168.5	41.32	175	145	93.54	188.3
**SD**	17.87	15.71	16.04	13.15	18.39	10.26	16.38	10.75	10.56	14.07	12.64	10.06	13.92	17.8	12.35	14.07	17.53	9.863
**SEM**	2.527	2.221	2.268	1.86	2.6	1.451	2.316	1.521	1.493	1.989	1.787	1.422	1.969	2.517	1.746	1.99	2.479	1.395
**CV%**	9.831	62.28	8.860	6.429	21.27	4.901	13.57	5.592	4.787	19.14	6.706	4.458	8.264	43.07	7.057	9.708	18.74	5.237
L 95% CI	176.7	20.76	176.5	200.8	81.21	206.5	116.0	189.2	217.5	69.50	184.8	222.7	164.5	36.26	171.5	141.0	88.56	185.5
U 95% CI	186.8	29.68	185.6	208.3	91.67	212.3	125.4	195.4	223.5	77.50	192.0	228.4	172.5	46.38	178.5	149.0	98.52	191.1

The background colors represent the average shade on each region.

**Table 2:** Qualitative assessment of the mucus synthesized into the mucosa.

	The Most Intense (Darkest) Color from the Average (Min Value)	Average Color Value	The Least Intense (Lightest) Color from the Average (Max Value)
PAS reaction			
The apical half of the glands in the mucosa	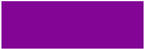	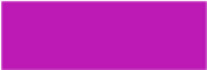	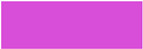
The basal half of the glands in the mucosa	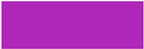	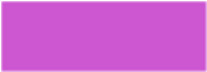	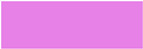
AB pH-2.5 staining			
The apical half of the glands in the mucosa	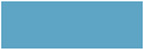	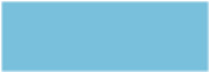	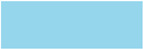
The basal half of the glands in the mucosa	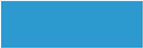	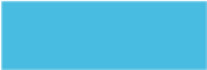	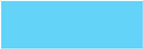
PAS-AB staining			
The apical half of the glands in the mucosa	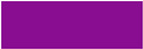	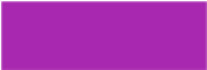	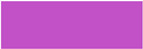
The basal half of the glands in the mucosa	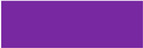	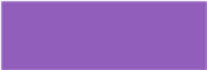	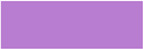

## Data Availability

The data supporting reported results can be found in the Supplementary Materials.

## Supplementary Materials

The following supporting information can be downloaded at: https://www.mdpi.com/article/10.3390/life15111716/s1.
