# Supplementary material for: New Insights into the Mucus-Secreting Cells in the Proventriculus of 10 Day Old Ross 308 Broiler Chickens—A Qualitative RGB Color Study by Histochemical Assessment
Source: Life (Basel). 2025 Nov 6;15(11):1716. doi: 10.3390/life15111716 (PMC12653235; doi:10.3390/life15111716)
Supplement: Supplementary file 1 [file life-15-01716-s001.zip › life-3932292-supplementary.pdf]

Supplementary file

RGB measurements

PAS						AB						PAS-AB					
Apical half			Basal half			Apical half			Basal half			Apical half			Basal half		
R	G	B	R	G	B	R	G	B	R	G	B	R	G	B	R	G	B
165	12	162	200	57	198	150	188	197	63	200	232	176	81	185	155	84	190
180	20	172	191	63	199	128	187	203	56	190	228	157	36	163	158	95	184
186	14	184	187	76	191	106	186	211	63	178	223	166	26	161	157	73	195
180	11	172	203	89	210	126	184	222	81	187	229	167	48	178	160	88	198
161	10	163	190	74	209	95	189	225	50	188	217	191	57	188	158	89	195
199	58	196	175	93	201	120	193	226	85	185	221	193	70	197	120	40	161
191	25	185	197	61	207	150	208	232	99	180	209	170	46	166	127	79	179
191	27	186	203	67	201	97	197	213	67	174	210	162	42	175	121	87	164
210	44	202	200	79	206	137	201	226	94	169	211	154	33	162	165	97	198
186	25	181	218	80	217	115	180	202	75	154	210	162	66	191	151	87	181
187	36	193	202	98	213	106	171	209	61	181	215	173	59	173	146	68	180
176	13	168	190	68	203	103	180	224	82	182	218	170	51	169	154	98	185
181	20	178	198	124	223	109	194	223	80	180	214	136	15	144	156	63	180
210	45	209	210	68	216	141	214	233	63	169	217	158	69	185	168	106	192
180	29	186	217	115	227	106	181	210	84	187	228	142	13	157	168	115	195
176	22	172	218	79	204	109	200	229	51	179	216	180	57	173	140	100	189
173	34	177	204	96	207	101	164	205	65	188	221	137	29	164	184	104	202
192	28	185	198	94	215	93	191	230	87	196	235	173	48	176	155	116	195
163	7	166	225	128	221	95	204	224	68	186	224	147	26	157	143	97	187
189	8	183	231	102	231	132	212	235	93	198	243	164	31	172	136	66	178
155	15	155	228	99	228	146	196	221	72	173	229	162	26	168	147	68	185
176	26	176	204	90	203	141	185	210	70	184	218	183	73	187	128	79	188
159	12	154	204	86	206	116	189	232	55	198	232	194	46	196	149	79	193
179	17	178	194	115	194	120	193	226	47	175	220	178	49	178	131	105	196
179	22	187	193	100	207	134	189	228	45	193	229	179	73	199	141	91	190
161	13	163	206	75	217	111	197	230	69	209	245	167	36	178	150	104	202
176	18	177	206	82	214	109	179	213	50	175	207	168	37	175	130	83	175
211	78	206	209	79	213	114	205	224	76	201	233	175	25	168	148	85	189
186	20	187	225	101	221	124	188	234	92	205	247	161	49	187	157	89	208
151	13	161	193	77	202	120	182	207	95	194	226	150	20	168	148	121	202
192	32	202	215	81	212	149	190	208	58	207	239	156	26	174	150	67	197
188	35	187	198	45	197	135	194	208	74	195	226	153	17	161	131	107	183
162	22	171	203	93	206	134	211	229	90	209	233	187	60	193	131	99	182
217	50	215	213	106	212	136	202	226	73	171	210	181	75	185	162	110	195
166	16	149	198	77	208	118	184	210	62	184	231	169	41	160	143	103	191
176	23	173	180	86	196	96	207	235	61	190	230	170	25	164	124	84	181
189	38	195	204	111	225	96	198	236	81	209	244	178	30	180	138	115	185
200	54	201	187	93	203	117	200	234	76	187	232	190	52	187	140	93	185
203	35	193	204	100	215	132	208	234	87	204	237	166	18	168	139	117	182
210	57	216	194	67	200	117	185	220	91	170	213	176	35	189	142	86	177
172	8	180	218	91	220	145	194	224	94	171	215	167	28	171	155	124	200
190	26	185	183	65	185	137	177	202	79	194	225	186	59	190	124	96	183
170	9	173	200	40	190	102	177	209	89	211	235	161	24	156	145	113	180

130	8	153	227	81	214	128	193	225	66	194	233	165	33	170	159	109	206
177	29	175	218	90	211	120	189	222	74	194	229	192	33	187	128	81	177
215	33	196	228	100	223	113	204	223	85	199	226	158	27	165	120	87	178
179	8	184	218	89	215	137	189	213	66	193	225	162	26	172	137	93	191
162	12	161	210	102	211	130	190	216	71	199	234	160	29	169	145	109	199
179	5	178	211	88	205	130	196	220	77	191	228	172	58	179	140	106	193
201	39	200	201	102	218	109	200	229	83	192	225	181	33	189	144	122	195
